# How to … Do Feasibility, Acceptability and Potential Effectiveness Studies in Health Professions Education Research

**DOI:** 10.1111/tct.70553

**Published:** 2026-10-29

**Authors:** John Sandars

**Affiliations:** ^1^ School of Medicine, Faculty of Health, Social Care and Medicine Edge Hill University Ormskirk Lancashire UK

## Abstract

New interventions in health professions education (HPE) are often widely disseminated before their feasibility, acceptability and potential effectiveness have been adequately evaluated. This creates uncertainty for educators and curriculum decision‐makers about whether these interventions have promise for progression, with adoption or adaptation and further evaluation into their own educational setting. This How to … article provides a practical guide to designing and reporting feasibility, acceptability and potential effectiveness studies for early‐stage evaluation of new interventions in HPE.

## 1 Introduction

Health professions education (HPE) generates a continuous stream of innovations that lead to new educational interventions, reflecting educators' creative efforts to address challenges in teaching and learning. A major dilemma for educators and curriculum decision‐makers is that new interventions are often widely disseminated at an early stage through conference posters, short communications and innovation reports in peer‐reviewed journals [1]. This creates uncertainty for those considering whether, and under what conditions, a new intervention shows promise for progression, with adoption or adaptation and further evaluation into their own educational setting since there often has been limited evaluation about their feasibility, acceptability and potential effectiveness.

*A major dilemma for educators and curriculum decision‐makers is that new interventions are often widely disseminated at an early‐stage.*

Evaluation of feasibility, acceptability and potential effectiveness is complicated because of conceptual confusion between the main types of early‐stage evaluation studies (proof‐of‐concept, feasibility and pilot studies), with inconsistent use of the terms and considerable overlap between the types of study purposes [2]. For example, proof‐of‐concept studies seek to establish whether the ideas that stimulate innovation can be translated into a practical new intervention; feasibility studies examine whether an intervention can be delivered successfully in the intended setting and pilot studies typically test the processes required for a subsequent definitive evaluation [2, 3, 4]. Consequently, it has been recommended that explicitly evaluating feasibility, acceptability and potential effectiveness may provide a clearer and more transparent approach for conducting early‐stage studies of new interventions [2].

This *How to … article* presents a practical guide for all HPE educators conducting early‐stage evaluations of new interventions about how to design and report feasibility, acceptability and potential effectiveness studies in HPE research. The article will also be useful for informing HPE educators and curriculum for appraising the findings of feasibility, acceptability and potential effectiveness studies in HPE research.

## 2 The Design of Feasibility, Acceptability and Potential Effectiveness Studies

The aim of feasibility, acceptability and potential effectiveness studies is to help educators and curriculum decision‐makers determine whether an educational intervention shows promise for progression, with wider adoption or adaptation and further evaluation into their own educational setting [2].

*The aim of feasibility, acceptability, and potential effectiveness studies is to help educators and curriculum decision‐makers determine whether an educational intervention shows promise for progression.*

These early‐stage evaluation studies need to answer three essential questions:

- Can this new intervention be delivered as intended (feasibility)?
- Do learners and educators find the new intervention acceptable (acceptability)?
- Does the new intervention show early signs of achieving its intended learning outcomes (potential effectiveness)?

Recommendations for conducting these studies can be informed by integrating the extensive literature about proof‐of‐concept, feasibility and pilot studies.

### 2.1 Understanding an Intervention's Feasibility

Key dimensions to evaluate include:

- Retention and attrition: proportion of participants completing the study.
- Intervention adherence: extent to which participants follow the intervention.
- Fidelity: degree to which the intervention is delivered as designed.
- Practicality: time, cost, staffing and resource requirements.

### 2.2 Understanding an Interventions' Acceptability

Key dimensions to evaluate include:

- Perceived usefulness: alignment with needs and expectations of the users of the new intervention.
- Perceived ease of use: time, effort and cognitive load required to use the new intervention.
- Willingness to participate: continuation rates and intention to continue use of the new intervention.

### 2.3 Understanding an Intervention's Potential Effectiveness

Key dimensions to evaluate include the acquisition of new knowledge and skills, often by post‐intervention measures. Knowledge and skills are fundamental for the development of new behaviours and performance and can also provide an indication that the new intervention has promise, but evaluation of behaviour and performance (and also transfer to practice) is more appropriate for larger and later‐stage impact evaluations [5].

## 3 Sample Size and Progression Criteria for Feasibility, Acceptability and Potential Effectiveness Studies

There is no universally accepted sample size for feasibility, acceptability and potential effectiveness studies, but it should be justified according to the information required to address the study objectives and make a progression decision. It has been recommended that essential information can be obtained with approximately 12 participants [6], but 20–50 participants have been recommended as being more appropriate for more precise estimates of feasibility, acceptability and potential effectiveness [7].

Pre‐specified progression criteria that define what constitutes sufficient information to adopt, adapt or reject a new intervention have been recommended [8]. Criteria may address recruitment, retention, completion, fidelity, acceptability and preliminary outcomes. A traffic‐light approach (green = adopt; amber = adapt; red = reject) provides a transparent framework for decision‐making, with thresholds justified by the priorities of educators and curriculum decision‐makers considering the promise of the new intervention rather than as universal standards.

## 4 Methods for Evaluating Feasibility, Acceptability and Potential Effectiveness

A mixed‐methods approach, combining quantitative indicators of recruitment, retention, completion, fidelity and preliminary outcomes with qualitative exploration of participants' experiences, acceptability, barriers, facilitators and contextual influences is recommended [2]. Qualitative data can be collected using interviews, focus groups and observations. Validated questionnaires can complement the measures, such as the Acceptability of Intervention Measure (AIM), Intervention Appropriateness Measure (IAM) and Feasibility of Intervention Measure (FIM) [9]. See Box 1.

Box 1 **Questionnaires for feasibility, acceptability and appropriateness [9].**

**Table:** 

1 Feasibility of Intervention Measure (FIM) The questions are: This intervention seems practical. This intervention seems realistic. This intervention seems implementable. This intervention seems doable. 2 Acceptability of Intervention Measure (AIM) The questions are: This intervention meets my approval. This intervention is appealing to me. I like this intervention. I welcome this intervention. 3 Intervention Appropriateness Measure (IAM) The questions are: This intervention seems fitting. This intervention seems suitable. This intervention seems applicable. This intervention seems like a good match. The term ‘This intervention’ can be replaced with the name of the specific educational innovation, such as ‘This simulation‐based educational intervention …’ rather than asking participants to rate a generic ‘intervention’.

## 5 Equity in Feasibility, Acceptability and Potential Effectiveness Studies

Equity is an essential consideration when conducting feasibility, acceptability and potential effectiveness studies since these dimensions are influenced by several factors, both individual and systemic [10]. For example, a new intervention can unintentionally disadvantage students who lack personal technology devices or flexible time to access online learning materials, making the intervention harder for them to fully participate. Evaluating feasibility requires checking whether some groups of learners need more support, whether educators can deliver the extra support, and whether adaptations are possible for learners with specific needs. Acceptability also needs to be assessed separately for different groups since what is considered to be useful or easy to use for one group of learners may not be so for another. Collecting data that has a focus on equity helps ensure a new intervention is fair for everyone.

*Equity is an essential consideration when conducting feasibility, acceptability and potential effectiveness studies.*

## 6 Reporting Feasibility, Acceptability and Potential Effectiveness Studies

There is often a lack of clear reporting of studies that evaluate feasibility, acceptability and potential effectiveness in HPE [11], but clear reporting enhances transparency and reproducibility to inform progression with wider adoption or adaptation and further evaluation into their own educational setting, and it also strengthens the credibility of this type of early‐stage research study.

It is essential to clearly report the new intervention so that educators and curriculum decision‐makers can understand exactly what was done, why it was done, and how it was delivered. Without transparent reporting, educators and curriculum decision‐makers cannot determine whether a new intervention should be progressed, with wider adoption or adaptation and further evaluation into their own educational setting. Researchers may consider the use of TIDieR (Template for Intervention Description and Replication) [12]. This is a 12‐item reporting checklist designed to improve the completeness and clarity with which interventions are described in research publications. See Box 2.

Box 2 **The main dimensions of Template for Intervention Description and Replication (TIDieR) [6].**

**Table:** 

Brief name—the name or label of the intervention Why—the rationale, theory or goal underpinning the intervention What (materials)—physical or informational materials used What (procedures)—procedures, activities and processes involved Who provided—expertise, background and training of intervention providers How—mode of delivery Where—location and relevant infrastructure When and how much—number of sessions, schedule, duration, intensity Tailoring—planned personalisation or adaptation Modifications—changes made during the study How well (planned)—fidelity assessment plans and strategies How well (actual)—fidelity results and extent of adherence

In addition, it is essential to clearly report the study design and to demonstrate that the study was systematically designed, conducted, analysed and reported so that readers can judge the credibility and trustworthiness of the findings.

## 7 Common Challenges When Conducting Feasibility, Acceptability and Potential Effectiveness Studies

There are several common challenges when conducting early‐stage evaluations of new interventions:

- Conceptual ambiguity and cautious interpretation of preliminary outcomes. Feasibility, acceptability and potential effectiveness studies are conducted to evaluate the extent that a new intervention is acceptable to learners and educators and feasible to deliver and show promising early outcomes, although there is still insufficient evidence to conclude that it is effective [2]. Effectiveness studies require appropriate rigorous experimental study designs with larger numbers of participants and across a variety of settings.
- Insufficient description of the new intervention and study design. Important factors such as intervention components, delivery processes, organisational constraints, educator readiness, available resources and learner variability should be reported systematically and frameworks such as TIDieR can support comprehensive intervention description. In addition, the study design should be clearly reported, including the sample size, methods of data collection and analysis.
- Ensuring stakeholder engagement and co‐design of the study. Evaluations should be designed around its intended users and their use of the information [13]. This ensures that educators and curriculum decision‐makers who have to make a judgement about whether to progress with wider adoption or adaptation and further evaluation into their own educational setting are presented with the most appropriate information that is required for making the judgement.

## 8 Illustrative Examples of Feasibility, Acceptability and Potential Effectiveness Studies in HPE

Two illustrative examples of feasibility, acceptability and potential effectiveness studies in HPE are provided: One study was a study of the feasibility, acceptability and potential effectiveness of an in situ cardiac arrest simulation involving both clinical and non‐clinical staff in general practice [14] (see Box 3) and one is a study of the feasibility, acceptability and potential effectiveness of teaching anatomy using virtual reality (VR) [15] (see Box 4).

Box 3 **Illustrative example A: Feasibility, acceptability and potential effectiveness.**

**Table:** 

Halls et al. [14] conducted a study of the feasibility, acceptability and potential effectiveness of an in situ cardiac arrest simulation involving both clinical and non‐clinical staff in general practice. Feasibility The study demonstrated that the simulation could be implemented within real general‐practice environments. Three practices successfully delivered the approximately 20‐min simulation to nine staff members in each practice followed by a 45‐min debrief, using each practice's own equipment and environment. However, practical challenges were identified, including staff availability, closing the practice, familiarity with equipment and the need for flexible approaches to involving staff. Acceptability Despite considerable apprehension before participation—including concerns about being assessed and filmed—participants generally regarded the simulation as a valuable learning experience. They particularly valued the realism of the scenario, use of their own equipment, participation of clinical and non‐clinical staff and opportunity to practise teamwork. Potential effectiveness The study did not directly establish educational or patient outcomes but identified promising indications of potential benefit. Participants reported improved awareness of team roles, opportunities to identify strengths and areas for improvement, greater familiarity with emergency equipment and changes to subsequent training and workplace practice. All three practices successfully managed the simulated emergency, and participants perceived benefits for teamwork and preparedness.

Box 4 **Illustrative example B: Feasibility, acceptability and potential effectiveness.**

**Table:** 

Walsh et al. [15] conducted a study of the feasibility, acceptability and potential effectiveness of teaching anatomy using virtual reality (VR). Feasibility A 30‐min guided VR anatomy session was successfully delivered to all 38 students using a VR headset and anatomy software. Students generally found the technology easy to use (mean 5.95/7), with 95% reporting that the software was easy to use. A small proportion (8%) experienced motion sickness, identifying an important implementation issue to address. Acceptability Acceptability was high: All students enjoyed the experience, 98% reported that they could interact with the 3D environment, all considered VR useful, and all indicated that they would use VR again. Potential effectiveness Students perceived that VR enhanced their understanding of anatomy, with all participants agreeing that it enhanced their understanding (mean of 6.58/7). However, learning effectiveness was not directly tested: There were no objective measures of learning, examination performance or knowledge retention.

## 9 Conclusion

Feasibility, acceptability and potential effectiveness studies are essential for evaluating the promise of new HPE interventions. The aim of feasibility, acceptability and potential effectiveness studies is to help educators and curriculum decision‐makers determine whether an educational innovation shows promise as a new intervention, with information needed to make a decision about whether, how and under what conditions an innovation should progress to wider adoption or adaptation and further evaluation into their own educational setting. The use of a systematic approach for the design and reporting of an early‐stage study can ensure that information disseminated to educators and curriculum decision‐makers is adequate for their needs.

## Author Contributions

**John Sandars:** conceptualisation, writing – original draft, writing – review and editing.

## Funding

The author has nothing to report.

## Conflicts of Interest

The author declares no conflicts of interest.

## Data Availability

Data sharing not applicable to this article as no datasets were generated or analysed during the current study.
